# Cardiac Cell Therapy: Insights into the Mechanisms of Tissue Repair

**DOI:** 10.3390/ijms22031201

**Published:** 2021-01-26

**Authors:** Hsuan Peng, Kazuhiro Shindo, Renée R. Donahue, Ahmed Abdel-Latif

**Affiliations:** Gill Heart and Vascular Institute and Division of Cardiovascular Medicine, University of Kentucky and The Lexington VA Medical Center, Lexington, KY 40508, USA; Hsuan.peng@uky.edu (H.P.); kazuhiro.shindo@uky.edu (K.S.); renee.donahue@uky.edu (R.R.D.)

**Keywords:** mesenchymal stem cells, stem cell therapy, myocardial infarction, inflammation, heart failure

## Abstract

Stem cell-based cardiac therapies have been extensively studied in recent years. However, the efficacy of cell delivery, engraftment, and differentiation post-transplant remain continuous challenges and represent opportunities to further refine our current strategies. Despite limited long-term cardiac retention, stem cell treatment leads to sustained cardiac benefit following myocardial infarction (MI). This review summarizes the current knowledge on stem cell based cardiac immunomodulation by highlighting the cellular and molecular mechanisms of different immune responses to mesenchymal stem cells (MSCs) and their secretory factors. This review also addresses the clinical evidence in the field.

## 1. Introduction

Advanced cardiac support, medical therapy and early reperfusion strategies have dramatically improved the survival rate in patients suffering from acute myocardial infarction (MI) [1]. Despite this success, the risk of heart failure (HF) following myocardial infarction remains high in these patients, and there are no effective treatments available to prevent this progression [2,3]. MI causes the loss of up to 1 billion cardiomyocytes [4]. Since the myocardium has negligible endogenous regenerative capacity, the significant loss of cardiomyocytes ultimately leads to formation of scar, altered cardiac structure, and compromised cardiac function. Development of HF following MI is closely associated with adverse cardiac remodeling, a process linked to worsening cardiac function and chamber dilatation [5]. While the extent of initial insult correlates with the level of post-MI remodeling, it is also highly dependent on the systemic immune response and ensuing cardiac inflammatory response [2,3]. In fact, the inflammatory response after MI dictates the degree of cardiac recovery. Inflammation, orchestrated by immune cells, is responsible for clearing dead cells and matrix debris at the injury site. This process is vital to subsequent tissue repair as it provides key molecular signals for activation of reparative processes. However, prolonged tissue inflammation and infiltration of pro-inflammatory cells in the post-MI myocardium exacerbates damage [6].

Cardiac repair is tightly coupled with the post-injury inflammatory process, which suggests that targeting inflammation may hold promise in preserving cardiac tissue and reducing mortality in patients surviving MI. Modulating the post-MI inflammatory response as a therapeutic intervention is supported by seminal observations of biological processes and cellular responses to tissue injury. As Huang et al. point out, modulating inflammation during the early phase post-MI prevents infarct expansion by reducing border zone cardiomyocyte (CM) injury and necrosis [7]. Attenuation of excessive and prolonged pro-inflammatory signaling may also protect cardiomyocytes during the cardiac remodeling phase by reducing apoptosis. In addition, modulation of the post-MI immune response could promote a healing microenvironment and reduce scar formation as the signaling pathways between inflammation, protease activation, and fibrogenic signaling are extensively connected. Lastly, selective activation of the immune signaling pathway could alter cell recruitment into the area of infarction. However, clinical studies on broad immunosuppressive agents for post-MI heart failure or other cardiac diseases do not improve cardiac healing [8,9]. Glucocorticosteroids and non-steroidal anti-inflammatory therapeutics are associated with higher mortality and recurrent MI [10,11], while studies aiming to deplete inflammatory cells fail to demonstrate benefit [12]. Therefore, strategies that modulate immune cells and their secretome after MI, rather than a complete inflammation blockade, may provide better therapeutic strategies.

Among the various immune cells involved in the post-MI immune response, neutrophils and macrophages play key role in the healing process. Neutrophils are the first immune cells to arrive at the injured myocardium and play an important role in the clearance of dead cells, in addition to setting the intensity of the subsequent inflammatory response. Macrophages also play an important role in the early inflammatory and subsequent reparative phases. They are generally classified into pro-inflammatory/classically activated/M1-like and anti-inflammatory/alternatively activated/reparative/M2-like macrophages based on gene profile and function [13,14]. Pro-inflammatory macrophages dominate the early phase after MI (1–5 days), while anti-inflammatory macrophages are in the majority in the later healing phase, promoting tissue healing and angiogenesis [15]. There is a growing body of evidence that suggests that alternative macrophage polarization to an anti-inflammatory phenotype protects against the early development of ischemic damage and subsequent adverse cardiac remodeling [16,17]. Given the complexity of immune signaling cascades and their interconnected biological functions, current approaches are refined to focus on targeting the distinct regulatory mechanisms that can direct specific immune cell populations to promote cardiac repair.

## 2. Stem Cells as Cardiac Immunomodulatory Therapy

Adult stem cell based cardiac therapies have been investigated in clinical trials with the rationale that they could repopulate and regrow/repair the lost/injured cardiac tissue [18]. However, the results from multiple studies indicate that transplanted adult stem cells do not directly replace the lost myocardium, they do not differentiate into cardiomyocytes. Instead, recent reports suggest that the observed cardiac functional improvement is associated with stem cell-mediated reparative mechanisms and immunomodulatory functions within the infarcted myocardium [19]. In-depth mechanistic studies further reveal that the stem cell secretome is enriched in various growth factors, cytokines, microRNAs, and exosomes that modify surrounding cells and the microenvironment. Increasing evidence suggests that stem cells orchestrate a pro-regenerative microenvironment in post infarcted tissue by modulation of specific immune pathways and cell populations. Among the stem cell types in the literature, bone marrow derived mesenchymal stem/stromal cells (MSCs) and cardiosphere-derived cells (CDCs) are most widely studied [20].

### 2.1. Immunoregulatory Function of Mesenchymal Stem/Stromal Cells (MSCs)

MSCs represent a multi-potent population is extensively investigated as cell therapy for multiple diseases due to their accessibility. MSCs are harvested from various adult tissues such as bone marrow, adipose tissue, connective tissues, and umbilical cord [21]. Unlike hematopoietic stem cells, MSCs are characterized by the presence of certain criteria including: (1) their ability to adhere to plastic culture plates under standard culture conditions; (2) the expression of CD105, CD73, CD44, and CD90 among others; (3) the lack of CD45 or CD11b expression; and (3) their ability to differentiate into osteoblasts, adipocytes, and chondroblasts in vitro [21].

While MSCs were initially examined in cardiac applications due to their capacity to differentiate into different cell types, It was later found that MSCs produce abundant paracrine factors including soluble growth factors, cytokines, and exosomes that mediate wound healing [21]. In addition, these secretory factors modulate the immune system both locally and systemically [22]. Together, these characteristics make MSCs a particularly attractive therapeutic cell type for post-MI cardiac applications. MSCs modulate inflammation by shifting leukocyte function and phenotypes via both direct cell contact and through soluble factors [23,24]. Different studies show that MSCs modulate neutrophil activity, regulate T-cell proliferation, and influence macrophage polarization. Here we focus on the multifaceted roles of MSCs in modulating immune cells in the context of cardiac injury.

#### 2.1.1. Immunoregulatory Effects of MSCs on Neutrophils

Following MI, damage-associated molecular patterns (DAMPs) activate tissue-resident cells to release pro-inflammatory cytokines and chemokines. This attracts a massive infiltration of the earliest immune cells to arrive in the myocardium after MI, neutrophils, to the cardiac tissue and initiates the deleterious inflammatory response [25]. In response to tissue injury, neutrophils undergo an oxidative response that triggers neutrophil extracellular trap (NETs) formation, neutrophil apoptosis and reactive oxygen species (ROS) production. Research shows that MSCs can suppress neutrophil activation and attenuate neutrophil mediated tissue injury by altering neutrophil oxidative metabolism [26,27]. Jiang et al., report that MSCs suppress the formation of NETs and the release of neutrophil death related protease by upregulating extracellular superoxide dismutase 3 (SOD3) in vivo [26]. In addition, IL-6 secretion from MSCs significantly inhibits neutrophil apoptosis in vitro [28]. Interestingly, this anti-apoptotic MSC activity does not require cell-to-cell contact, as the cells cultured in trans-well experiments still produce this effect. Further research shows that MSCs have no effect on neutrophil phagocytosis, expression of adhesion molecules, or chemotaxis [29].

#### 2.1.2. Immunoregulatory Effects of MSCs on Macrophage

Since macrophages are key mediators of immune response, the effects of MSCs on macrophage populations after cardiac injury are well documented in many studies and represent a critical step in MSC-mediated cardiac protection [30]. In co-culture experiments, MSCs reduce macrophage production of key pro-inflammatory cytokines such as TNFα, IL-1β, IL-6, IFNγ, and IL-12; while simultaneously increasing anti-inflammatory cytokine expression [31]. MSC secretomes enriched with Prostaglandin E2 (PGE2), IL-1Rα, and TGFβ, are known to facilitate M1 to M2 macrophage polarization [32], In addition, MSCs recruit macrophages to the infarct site and induce M2 macrophage polarization through the release of IL-10, indoleamine 2,3-dioxygenase (IDO), C-C motif ligand 18 (CCL18) and PGE2 [33,34]. Interestingly, pro-inflammatory factors such as IFNγ, TNFα and LPS further enhance MSC-mediated M2 macrophage polarization by enhancing MSC production of PGE2, indoleamine 2,3-dioxygenase (IDO), and cyclo-oxygenase 2 (COX2) [35]. Macrophages play a key role in cardiac recovery post-MI as their phenotypes and secretomes closely govern multiple aspects of tissue recovery after injury [36]. By polarizing macrophages to an anti-inflammatory phenotype early after injury, MSCs may prevent a maladaptive immune response and contribute to multiple aspects of cardiac recovery.

#### 2.1.3. Immunoregulatory Effects of MSCs on Lymphocytes

The interaction between MSCs and lymphocytes is an active area of research. Studies show that MSCs have direct and indirect effects on the activation and proliferative state of lymphocytes. One proposed mediator of this phenomenon is an antagonist of IL1 receptor (IL1RA) secreted by MSCs. Luz-Crawford et al. report that MSC secreted IL1RA acts on both macrophages, by inducing a polarization toward the anti-inflammatory M2 phenotype; and on B-lymphocytes, by reducing plasmablast formation [37]. This is important in the context of MI since B-lymphocytes trigger monocyte mobilization and impair heart functional recovery after MI [38]. Moreover, B cells produce antibodies that interfere with cardiomyocyte function and are elevated in heart failure [39]. Although detailed mechanisms are still lacking, evidence suggests that MSC-derived CCL2 inhibits STAT3 activation in plasma cells, leading to attenuated immunoglobulin production [40]. Therefore, the data suggest an important and critical role for MSCs in modulating the function of lymphocytes following cardiac ischemic injury.

There is also considerable evidence to support MSC mediated T-cell modulation. A murine study utilizing CD4^+^ T cell-deficient mice resulted in significantly smaller infarct sizes compared with wild type controls This study is the first evidence that CD4^+^ T cells contribute to myocardial ischemia-reperfusion injury [41]. Thus, regulatory mechanisms that prevent T cell expansion after MI can directly reduce the production and infiltration of pro-inflammatory T cell populations and prevent excessive myocardial injury. Reports have identified that cell-to-cell interaction between MSCs and T cells promotes T cell apoptosis via co-inhibitory surface ligands, Fas ligand (FasL) and TNF-Related Apoptosis-Inducing Ligand (TRAIL) [42,43]. In addition, MSC secretomes enriched in inducible NO synthase (iNOS), IDO, TGFβ, and PGE2 mediate the phenotype, proliferation, and activation state of T cell populations without direct cell contact [27,42]. Furthermore, MSCs enhance the immunosuppressive capabilities of FOXP3^+^ T-regulatory cell populations, which further limits pro-inflammatory T cell proliferation [44], which is believed to be mediated by TGFβ and PGE2 enriched MSC secretome.

### 2.2. Immunomodulatory Properties of Cardiosphere-Derived Cells (CDCs)

Cardiosphere-derived cells (CDCs) are a cardiac-derived progenitor cell population that resemble the phenotype of MSCs and can be expanded ex vivo from cardiac tissue biopsies. There are multiple studies that examined resident cardiac progenitor cells such as cKit+ and Sca1+ cell populations. These studies reached similar conclusions and indicated that the contribution of these cells to adult myocardial repair and homeostasis is limited [18,45,46]. However, this methodology has been challenged by other investigators and this topic remains controversial [47,48]. Nonetheless, the consensus in the field is that the minimal intrinsic repair and homeostasis observed in adult mammalian heart is related to negligible proliferation of existing cardiomyocytes. Recent evidence has suggested a link between CDCs and the development if atrial myxomas [49], however, it is important to note that these observation has not been replicated in clinical studies that transplanted CSCs in multiple clinical scenarios. Studies show that CDCs possess stem cell properties including clonogenicity and multilineage differentiation, and have been shown to enhance cardiac repair in preclinical models [50]. In clinical trials, autologous CDCs have been proven safe for treating patients with MI [51,52]. Early research proposed that CDC-mediated cardiac repair mechanisms include direct differentiation and contribution to new myocardium, however, limited engraftment of injected cells suggests that paracrine effect is likely responsible for the majority of the observed therapeutic benefits [53].

Similar to MSCs, allogenic CDCs are reported to exert immunomodulatory effects. In a co-culture system, Dutton et al. show that MHC-mismatched CDCs suppress lymphocyte proliferation and activation in response to Concanavalin A [54]. The authors report that CDC mediated inhibition of lymphocyte proliferation is partially facilitated by soluble factors including high levels of PGE2, which leads to down-regulation of lymphocyte CD25 expression via the EP4 receptor. In the setting of MI, CDC secreted PGE2 can have a direct reparative effect by modulating cardiac inflammation.

Another major component of the CDC secretome includes exosomes, which reportedly mediate macrophage polarization [55]. Intracoronary injection of CDC derived exosomes (EV-CDCs) results in an increase of M2-like macrophages with lower pro-inflammatory gene expression [56]. Additional mechanistic studies demonstrate that EV-CDC-derived exosomes are enriched in regulatory peptides and miRNAs related to immune and cardiac function regulation [57]. Cambier et al. reported that EV-CDCs contain high levels of a Y RNA fragment (YF1) which induces macrophage IL-10 production. YF1 primed macrophages are also cytoprotective for CMs and in vitro reduce the detrimental effects of oxidative stress through secreting IL-10. Intracoronary administration of EV-YF1 following ischemia/reperfusion in rats reduces infarct size. In vivo studies in rats and pigs using EV-CDCs also show a reduction of infiltrating macrophages in the infarcted tissue. These observed changes are linked to EV-CDC mediated alternative macrophage polarization through miRNAs, such as mir-181b [58].

## 3. MSC Therapy in Clinical Trials

Various types of stem cells are utilized to enhance myocardial function in ischemic heart disease (IHD), yet the optimal cell type for cardiac repair is unknown. There are two main mechanisms attributed to stem cell-based therapies: the widely accepted paracrine mechanism through which cells release a myriad of growth factors that activate endogenous pathways, eventually aiding the ailing cardiac muscle; and the less proven theory of trans-differentiation into cardiac cells [59]. Mesenchymal stem cells (MSCs) emerge as a leading candidate for cardiac applications and exert the majority of their beneficial effects through paracrine pathways [60]. This part of the review focuses on the clinical applications of cell therapies in IHD with a particular focus on MSCs (Table 1 and Table 2).

### 3.1. Acute Myocardial Infarction (AMI)

Autologous bone marrow mononuclear stem cells (BMMNCs) are the most clinically utilized cell type for ischemic heart disease. Autologous BMMNCs offer multiple advantages including minimal ex-vivo processing, easy accessibility, and reduced immunogenicity. Initial results were promising, however, more careful examination of their benefits in recent trials demonstrates little improvement in cardiac function compared to standard therapy. Large-scale, randomized, placebo-controlled trials show only minor benefits, as confirmed by a meta-analysis published in 2015 [61,62]. In addition, the phase III BAMI trial announced in 2020, suffered slow enrollment and failed to demonstrate mortality benefit with BMMNCs [63].

Bone marrow mesenchymal stem cells (BMMSCs), on the other hand, fare better in animal and human studies. MSCs are rare and pluripotent cells that represent only 0.01% of total BMMNCs and can differentiate into a variety of mesoderm lines such as adipocytes, chondrocytes, and bone cells [64]. In 2004, Chen et al. report the first randomized trial to deliver autologous MSCs to post-AMI patients via intracoronary injection. Six months following therapy, the LVEF in the MSC group demonstrate significant improvement compared to standard therapy. This study also establishes the safety of autologous MSCs for cardiac applications [65]. Subsequently, Hare et al. report the safety and efficacy of intravenous administration of allogeneic MSCs in a post-primary percutaneous coronary intervention (PCI) AMI population. Similar to intracoronary administration, intravenous MSCs result in enhanced LVEF recovery and no side effects [66]. The beneficial effects of MSCs and their safety are confirmed in a study by Lee et al. who randomize 58 AMI patients to receive MSCs or placebo [67].

Adipose tissue-derived mesenchymal stem cells (ADMSCs) share many biological properties with BMMSCs and hence gathered interest for their use in cardiac applications [68]. In fact, the frequency of colony formation in ADMSCs exceeds that of BM and cord blood (CB) derived MSCs. This makes them an attractive target for tissue regeneration applications, since ADMSCs are expected to have the same therapeutic potential as BMMSCs but are more easily and safely collected [69]. In the APOLLO trial, Vulliet and colleagues randomize 14 patients with AMI to receive intracoronary infusion of ADMSCs or placebo and patients are followed for 6 months, after which cardiac perfusion and scar formation are assessed using single-photon emission computed tomography (SPECT). The study shows similar benefits to those observed with BMMSCs in enhancement of cardiac tissue perfusion and reductions in scar size [70].

MSCs from various compartments of the umbilical cord, such as veins, arteries, Wharton’s jelly, and umbilical cord lining, accumulate in damaged tissue and promote tissue repair. Umbilical cord- mesenchymal stem cells (UC-MSCs) have faster self-renewal capacity compared to BMMSCs and are less likely to form teratomas [71]. Gao et al. report the efficacy of UC-MSCs in 116 patients with ST elevation myocardial infarction (STEMI). UC-MSC treated patients have better cardiac functional recovery, ameliorated adverse cardiac remodeling and enhanced cardiac perfusion as assessed by SPECT 18 months after MI [72].

### 3.2. Chronic Heart Failure Reduced Ejection Fraction (Chronic HFrEF)

Multiple human studies examine MSC therapy in patients with chronic heart failure with reduced EF (HFrEF) including ischemic cardiomyopathy (ICM), dilated cardiomyopathy (DCM) and non-ischemic cardiomyopathy (NICM) (summarized in Table 1). In the POSEIDON trial, Hare et al. conduct a study comparing the safety and efficacy of allogeneic vs. autologous MSCs in ICM patients. The study demonstrates the comparable efficacy and safety of both approaches, yielding significant reduction in infarct size and improvement in cardiac functional recovery. The safety of both cell strategies is confirmed with no side effects attributed to either approach. However, in the autologous BMMSC-administered group, 6-min walk test is significantly increased as compared with allogeneic BMMSCs [73]. In a follow-up study using allogeneic BMMSCs in patients with NIDCM, BMMSC-treated patients experience smaller scar size and less heart failure symptoms at 12 months of follow-up [74]. These studies and others in the literature confirm the safety of BMMSCs in chronic HFrEF patients and suggest that allogeneic MSCs may be used as an alternative to autologous MSCs. However, these clinical trials generally enroll small numbers of study subjects and some of them lack a placebo arm, hence, future large randomized studies are needed to advance the field forward.

Several studies compare the effectiveness of BMMNCs and MSCs. The TAC-HFT trial (2014) is a phase I/II study of BMMNCs vs. BMMSCs or placebo in ICM patients with LVEF <50%. Patients with MSC treatment show significant improvements in symptoms, infarct size, and regional as well as global myocardial function [75]. These results suggest the benefit of using selected populations of BM cells for cardiac applications. In 2014, Ascheim et al. report a randomized clinical trial comparing allogeneic MSCs to placebo in advanced heart failure patients requiring left ventricular assist device (LVAD) support [76]. They report no clear advantage to MSC administration, although MSC-treated patients experience more successful LVAD weaning and longer duration off LVAD support. These results are replicated in multiple small clinical trials, which supports the clinical benefit of MSCs in comparison to standard care alone in cardiomyopathy patients in terms of both LVEF improvement and symptom reduction [77,78,79,80,81].

Preconditioning MSCs prior to transplantation incorporates the use of specific growth factors to enhance their cardioprotective effects, engraftment or survival after transplantation. The cardiogenic stem cell therapy (C-CURE) trial tests the utility of cardiogenic cocktails to enhance the therapeutic benefit of MSCs, including TGFβ, BMP4, activin-A, retinoic acid, FGF2, IGF1, alpha-thrombin, and IL-6, in addition to 5% platelet lysate at the onset of a 5-day incubation period. This plan was to induce cardiac transcription factor expression, potentiate nuclear translocation, and maintain cell cycle progression in an attempt to enhance the therapeutic effect of autologous MSCs for cardiac regeneration in ICM patients [82]. While they identify no adverse events with preconditioned autologous BMMSCs and report significant improvement in global left ventricular function, reduced adverse remodeling and increased 6-min walk test at 2 years follow-up, it is important to note that the C-CURE trial is a small clinical study which does not include a placebo arm. Bartunek et al. confirm the safety and efficacy of cardiac preconditioned autologous BMMSCs [84]. In CHART-1 trial, 271 patients with ischemic cardiomyopathy are randomized to receive either intracranial delivery of autologous cardiogenic preconditioned BMMSCs or sham procedure. The primary outcomes (all-cause mortality, worsening heart failure events, Minnesota Living with Heart Failure Questionnaire (MLHFQ), 6-min walk distance, LVEF and left ventricular end systolic volume (LVESV)) were similar between both groups. However, cell therapy improves 6-min walk distance in the subgroup of patients with baseline left ventricular end-diastolic volume (LVEDV) of 200–370 mL (60% of patients), which suggests a differential therapeutic response based on the degree of underlying cardiac dysfunction. While LVEDV was used to stratify cardiac dysfunction in the CHART-1 trial, other cardiac functional indices may further stratify patients and identify those with the highest likelihood of therapeutic benefit.

In addition to studies that focus on BMMSCs, Perin et al. examine the therapeutic utility of ADMSCs in ICM patients [83]. 27 patients with ICM are randomized to either ADMSCs or placebo and followed for 36 months. The change in exercise capacity from baseline to 6 and 18 months is significantly ENHANCED in ADMSC-treated patients compared with controls. In addition, ADMSC-treated patients show significant improvement in total left ventricular mass by magnetic resonance imaging and wall motion score index.

The RIMECARD trial examines the efficacy of UC-MSCs in HFrEF patients [85]. UC-MSC-treated patients show no adverse events related to the cell infusion and none of the patients develop alloantibodies to the UC-MSCs. Furthermore, UC-MSC-treated patients exhibit significant improvement in LVEF. In addition, the recently reported HUC-HEART trial compares the effects of UC-MSCs and BMMNCs in ICM patients [86]. UC-MSCs improve global cardiac function, stroke volume, and 6-min walk distance at 12-month follow-up. Improvements in infarct size are observed in both UC-MSC and BMMNC treated arms, but the improvement is greater in UC-MSC-treated patients.

These results suggest a high degree of safety of MSC therapy in patients with acute MI and chronic HFrEF including ICM and NIDCM. The efficacy of MSC therapy in human studies is consistent, yet minimal, and efforts are needed to further refine the therapy and its timing to uncover the full potential of this therapeutic. The phase 3 DREAM-HF trial of randomized allogeneic MSCs to 566 patients with chronic HF due to LV systolic dysfunction of either ischemic or nonischemic etiology is underway and these results may help identify patients and clinical scenarios where MSC therapy will be most beneficial [87].

## 4. Cardiosphere Derived Stem Cells (CDCs)

CDCs are extracted and isolated from the patient’s own myocardium providing an excellent source of autologous stem cells for cardiac applications. After early success in animal models, multiple clinical trials demonstrate the safety and efficacy of autologous CDC therapy in humans (summarized in Table 2). In the CADUCEUS trial, enrolling 25 patients (17 in CDC group and 8 in standard of care alone group) 2–4 weeks after myocardial infarction with LVEF 25–45%, autologous CDCs are infused into the infarct-related artery 1.5–3 months after myocardial infarction [51,52]. CDC therapy is safe and associated with significant reduction in scar mass as well as increases in viable cardiac mass, regional contractility, and regional systolic wall thickening. However, these positive changes do not result in significant improvement in LVEF and LVEDV at 6 months. The DYNAMIC trial, enrolling 14 patients with dilated cardiomyopathy (ischemic and nonischemic) with an EF < 35% and NNYHA class III/IV, delivers four escalating doses of allogenic CDCs by sequential non-occlusive technique to all three major coronary arteries [88]. No primary safety endpoints are observed, confirming the safety of therapy. Compared to baseline there is an improvement in EF, LVESV, quality of life (QOL) questionnaires and NYHA class at 6 months post therapy.

The second generation of CDC trials is larger and conducts more comprehensive studies. ALLSTAR trial enrolls 134 patients (90 to the CDC group and 44 to the placebo group), 4 weeks to 12 months after MI, with left ventricular ejection fraction (LVEF) ≤ 45% and LV scar size ≥15% of LV mass by cardiac MRI [89]. Allogenic CDCs are infused into the infarct-related artery. Intracoronary delivery of allogeneic CDCs is safe but is not associated with a reduction in LV scar size, the primary endpoint. The trial is subsequently discontinued due to the low probability of detecting a significant treatment effect of CDCs based on the primary endpoint. However, infusion of CDCs appears to have a modest favorable impact on LV remodeling, with halted increases in LVESV and LVEDV relative to placebo at 6 months. Additionally, there is a greater reduction in NT-proBNP values at 6 months in CDC-treated patients.

The difference between the two major CDC human trials is interesting. The CADUCEUS trial shows an improvement in scar size but the ALLSTAR trial does not confirm this finding. While the CADUCEUS trial demonstrates 7.7% reduction in scar size at 6 months with autologous CDC infusion and 0.3% reduction in scar size in controls [51], the ALLSTAR trial demonstrates a 5.0% reduction in scar size at 6 months with allogenic CDC and 4.1% reduction in scar size in controls [89]. The difference between these two trials is due to change in scar size in the control group in the latter study. This difference may be related to the patient population enrolled and recent advances in standard of care therapy.

## 5. Future Perspectives

Cardiac repair is a complex process with dynamic changes in tissue composition and cellular populations at play. Stem cell therapy offers a promising clinical treatment option to modulate post-injury inflammation and enhance cardiac recovery in a multitude of acute and chronic cardiac conditions. Early studies of cardiac cell therapy utilizing MSCs and CDCs successfully demonstrated their clinical safety and efficacy while highlighting important challenges that need to be addressed as the field continues to move forward.

First, in order to refine and maximize the benefits of stem cell based cardiac therapy, there is real need for improving our understanding of the biology behind MSC- and CDC-mediated immunomodulation. It has been shown that tissue source, culture conditions, isolation methods, and cell preservation methods influence MSC phenotype and secretory function [90,91]. Lack of standardized MSC isolation and culture protocols also complicate the evaluation of MSC based treatments [92]. Moving forward, it is particularly important to compare these methodologies and stanbdardize them for future cardiac regeneration studies. In addition, rigorous studies comparing MSCs-derived cellular products with intact MSCs are needed as recent evidence suggest that apoptotic, metabolically inactivated, or fragmented MSCs still possess immunomodulatory capabilities [93,94]. This approach provides valuable alternative to live MSC treatment, as cell fragments or byproducts carry reduced tumorigenic or cellular rejection concerns.

Second, an important part of cardiac cell-based therapies depends on their retention and ability to survive in the heart following transplantation. The incorporation of biomaterials such as biocompatible scaffolds are presently being investigated in animal models to enhance the survival of transplanted cells. Future studies focused on developing biodegradable and biocompatible scaffold materials with refined encapsulation methods are likely to enhance the efficacy of cell-based therapy while reducing the need for large number of transplanted cells.

Third, aging is one of the most significant risk factors in cardiovascular disease. While studies report that CDCs and MSCs are beneficial in young animal, there is limited evidence on whether these therapies improve aging related cardiac functional decline [95]. Since aging also affects the immune system at multiple levels, future studies need to address whether stem cell mediated immunomodulation slows the progression of age-related cardiac decline.

Lastly, the synergy of combining different progenitor cell types with complementary properties might lead to enhanced cardiac repair. For instance, since cardiac damage does not lead to the generation of new cardiomyocytes (CMs), induced pluripotent stem cells (iPSCs) derived CMs (iPSC-CMs) are an attractive therapeutic approach to replace lost cardiac muscle. New strategies combining three-dimensional (3D) cardiac organoids (CO) with iPSC-CMs show enhanced CM maturation over conventional two-dimensional (2D) culture systems [96]. Advances in this area offers an opportunity to transplant an organized mini-organ/tissue incorporating stem cells, vascular cells, and iPSC-CMs in a defined micro-anatomy. In addition to direct transplantation, COs offer the opportunity to personalize drug screening and disease modeling *in vitro*. However, no rigorous clinical studies on CO transplantation in humans are performed to date.

## 6. Conclusions

The very limited cardiac recovery capacity of the adult heart drives a large effort in recent decades to facilitate myocardial repair. MSCs and CDCs hold the therapeutic potential to orchestrate balanced cardiac healing by recruitment of immune cells to the injury site for necessary matrix remodeling and removal of dead cells while simultaneously modulating their phenotype to enhance tissue recovery and promote wound healing. While there is no clear evidence of trans-differentiation of transplanted cells into new cardiomyocytes, there is clear evidence of paracrine activity. Advancements in identification and isolation of specific stem cell paracrine factors that promote healing and reduce unchecked inflammation will lead to subsequent refinement of stem cell-based therapies, which remain a promising treatment for acute MI patients and other cardiovascular diseases. Future generations of refined stem cell-based therapies remain a promising treatment for acute MI patients.

## Figures and Tables

**Table 1:** Human studies of the cardiac applications of mesenchymal stem cells.

Study Name	Year	Disease	Study Type	Cell Type	n	Follow Up	Results
**AMI**							
Chen et al. [71]	2004	AMI	Randomized, PC	Autologous BMMSCs	69	6 m	Improved LVEF and myocardial perfusion
Hare et al. [72]	2009	AMI	Randomized, DB, PC	Allogeneic BMMSCs	53	6 m	Improved LVEF and symptoms
APOLLO [76]	2012	AMI	Randomized, DB, PC	Autologous ADSCs	14	6 m	Improved perfusion and scar formation
Lee et al. [73]	2014	AMI	Randomized, No PC	Autologous BMMSCs	58	6 m	Improved LVEF
Gao et al. [78]	2015	STEMI	Randomized, DB, PC	UC-MSCs	116	18 m	Improved LVEF, LV volumes, perfusion
**Chronic HFrEF**							
POSEIDON [73]	2012	ICM with LVEF < 50	Randomized, No PC	Autologous or Allogeneic BMMSCs	30	13 m	Autologous MSCs-improved 6 mw and reduced infarct size
							Allogenic MSCs-improved LVEDV and reduced infarct size
C-CURE [82]	2013	ICM with LVEF < 40,	Randomized, No PC	Autologous cardiac preconditioned BMMSCs	47	2 y	Improved LVEF, LVESV, and symptoms
		NYHA 2 or 3					
TAC-HFT [75]	2014	ICM with LVEF < 50	Randomized, DB, PC	Autologous BMMNCs or MSCs	65	12 m	MSCs-improved symptom, 6 mw, and reduced infarct size.
							BMMNCs-improved symptom and regional myocardial function
Ascheim et al. [76]	2014	Chronic HFrEF with LVAD	Randomized, DB, PC	Allogeneic BMMSCs	30	12 m	No significant difference between treatments
PRECISE [83]	2014	ICM with NYHA 2-3	Randomized, DB, PC	Autologous ADSCs	27	36 m	Preserved MVO_2_, improved LV mass and wall motion score index
PROMETHEUS [77]	2014	ICM, CABG	Randomized, No PC	Autologous BMMSCs	6	18 m	Reduced infarct size
MSC-HF [78]	2015	ICM with NYHA 2-3,	Randomized, DB, PC	Autologous BMMSCs	55	6 m	Improved LVEF and LV volumes, symptom
		LVEF < 45					
Perin et al. [79]	2015	Chronic HFrEF	Randomized, DB, PC	Allogeneic BMMSCs	60	3 y	Improved mortality and HF-related MACE
MESAMI1 pilot [80]	2016	ICM with LVEF < 35	No randomized	Autologous BMMSCs	10	2 y	Improved LVEF and LV volumes
CHART-1 [84]	2017	ICM with LVEF < 35,	Randomized, DB, PC	Autologous cardiac preconditioned BMMSCs	271	39 w	No significant different
		NYHA 2-4					(Patient with LVEDV 200-370mL-improved 6mw)
RIMECARD [85]	2017	Chronic HFrEF	Randomized, DB, PC	UC-MSCs	30	12 m	Improved symptoms and LVEF
		with NYHA 1-3, LVEF < 40					
POSEIDON-DCM [74]	2017	NIDCM with LVEF < 40	Randomized, No PC	Autologous or Allogeneic BMMSCs	37	12 m	Improved LVEF (Autologous vs. Allogenic: no significant difference) and 6 min walk test (Autologous better than Allogenic).
TRIDENT [81]	2017	ICM	Randomized, DB, PC	Allogeneic BMMSCs	60	12 m	Improved infarct size, symptom
HUC-HEART [86]	2020	ICM with CABG,	Randomized, SB, No PC	UC-MSCs or BMMNCs	46	12 m	UC-MSCs-improved EF, SV, 6 mw, and reduced infarct size
		LVEF < 45					BMMNCs–reduced infarct size

AMI: acute myocardial infarction, HFrEF: heart failure with reduced ejection fraction, STEMI: ST-elevation myocardial infarction, ICM: ischemic cardiomyopathy, LVEF: left ventricular ejection fraction, NYHA: New York heart association functional classification, LVAD: left ventricular assist device, CABG: Coronary artery bypass grafting, NIDCM: non-ischaemic dilated cardiomyopathy, PC: placebo control, DB: double blind, SB: single blind, BMMSCs: bone marrow mesenchymal stem cells, ADSCs: adipose tissue derived stem cells, UC-MSCs: Human umbilical cord-derived mesenchymal stem cells, BMMNCs: Bone marrow-derived mononuclear cells, LV volumes: left ventricular volumes, 6 mw: 6 min walk test, LVEDV: left ventricular end-diastolic volume, MVO_2_: myocardial volume oxygen_,_ HF-related MACE: heart failure-related 3-major adverse cardiovascular events, SV: stroke volume.

**Table 2:** Human studies of the cardiac applications of cardiosphere derived stem cells.

Study Name	Year	Disease	Study Type	Cell Type	n	Follow Up	Results
CADUCEUS [51,52]	2014	ICM	Randomized, No PC	Autologous CDCs	25	1 y	Improved scar size, viable heart mass, regional contractility, regional systolic wall thickening
DYNAMIC [88]	2020	HFrEF	No Randomized	Allogeneic CDCs	14	1 y	Improved LVEF, QOL
ALLSTAR [89]	2020	ICM	Randomized, DB, PC,	Allogeneic CDCs	134	6 m	Improved LVEDV, LVESV, decreased plasma BNP

ICM: ischemic cardiomyopathy, HFrEF: heart failure with reduced ejection fraction, PC: positive control, DB: double blind, CDCs: cardiosphere-derived cells, LVEF: left ventricular ejection fraction, QOL: quality of life, LVEDV: left ventricular end-diastolic volume, LVESV: left ventricular end-systolic volume.
